# Reflection confocal microscope characteristics of rose acne and some thoughts caused by it

**DOI:** 10.1111/srt.13192

**Published:** 2022-07-05

**Authors:** Hongyong Sun, Yan Duan, Ruiya Li

**Affiliations:** ^1^ Department of Dermatology People's Hospital of Inner Mongolia Autonomous Region Hohhot China

Dear Editor,

Recently, in clinical work, we found some common skin CT features of rose acne by examining some patients with a clinical diagnosis of “Rose acne” with a reflective confocal microscope (skin CT) and collected and summarized these features, hoping to provide more objective basis for the diagnosis and identification of rose acne. In addition, in the process of analyzing the skin CT features of patients with rosacea, it also triggered some thoughts. Now I will share details with you.

Rosacea is a chronic recurrent inflammatory disease that often occurs in the middle of the face and mainly involves facial blood vessels, nerves, hair follicles and sebaceous units.^1^ The main clinical manifestations are paroxysmal flushing of facial skin, persistent erythema or papule, pustule, telangiectasia and so on (Figure 1A,B). A few patients may have hyperplasia, hypertrophy and eye changes. At present, the diagnostic criteria of rose acne have been relatively standardized and mature, but most of them are based on clinical subjective judgment. There are few reports on the research and exploration of skin CT features of rose acne.

**FIGURE 1:**
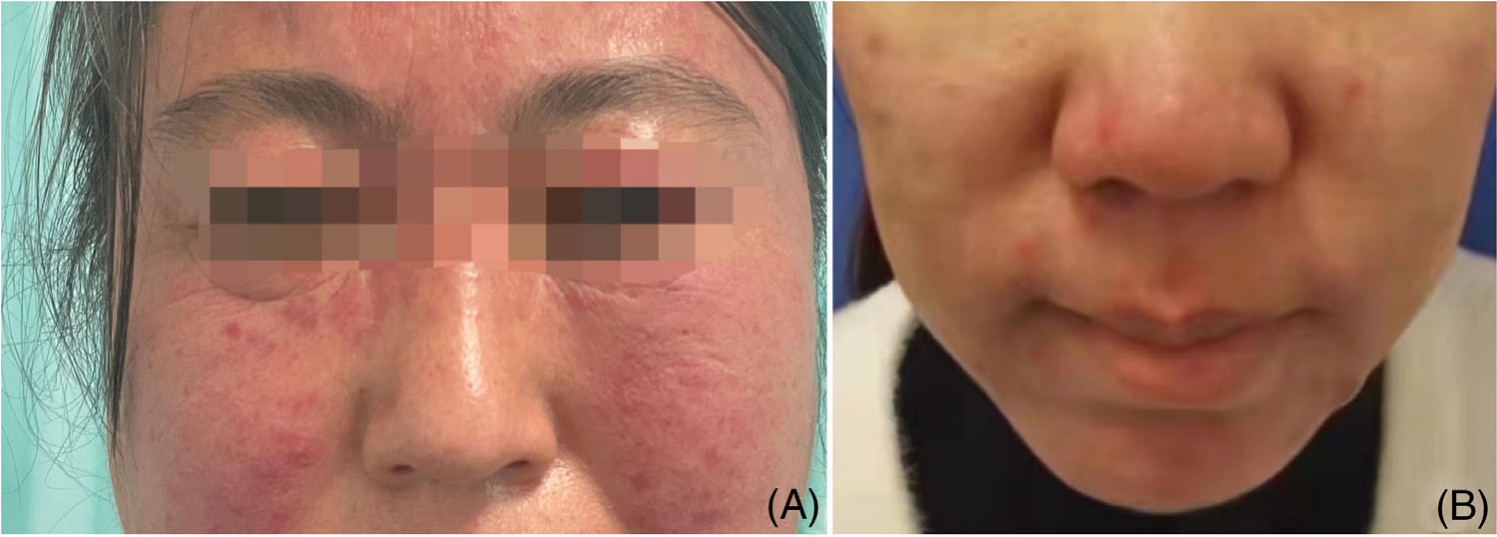
(A) Clinical picture of rosacea 1; (B) clinical picture of rosacea 2.

Reflective confocal microscope, also known as skin CT, is a skin image diagnosis technology rising in recent years. It can detect patients’ skin lesions in “noninvasive, real‐time and in vivo,”^2^ collect and analyze cross‐sectional imaging images of skin lesions, and use them for disease diagnosis and condition evaluation. In our clinical work, we collected the skin CT images of patients with rosacea and found that their skin CT features have similarities. The main manifestations are as follows: (1) The epidermis atrophied and flattened, and the structure of dermal papillary ring, was not obvious (Figure 2A); (2) spongy edema with different degrees can be seen in the spinous layer, showing a liquid dark area (Figure 2B); (3) the unit diameter of hair follicle sebaceous gland increases, the infundibulum of hair follicle expands, and some horny like substances are filled to form hair follicle horn plugs and the refractive index increases (Figure 2C); (4) some perifollicular abscesses are formed, and high refraction neutrophils can be seen (Figure 2D); (5) the blood vessels around the hair follicle and the superficial and middle dermis were significantly proliferated, dilated and congested, with abundant blood supply (Figure 2E); (6) more inflammatory cell infiltration can be seen around blood vessels and hair follicles, some cells are large and slightly irregular, and the refractive index is high (Figure 2F); (7) some patients may have Demodex structures colonized in hair follicle sebaceous gland units^3^ (Figure 2G).

**FIGURE 2:**
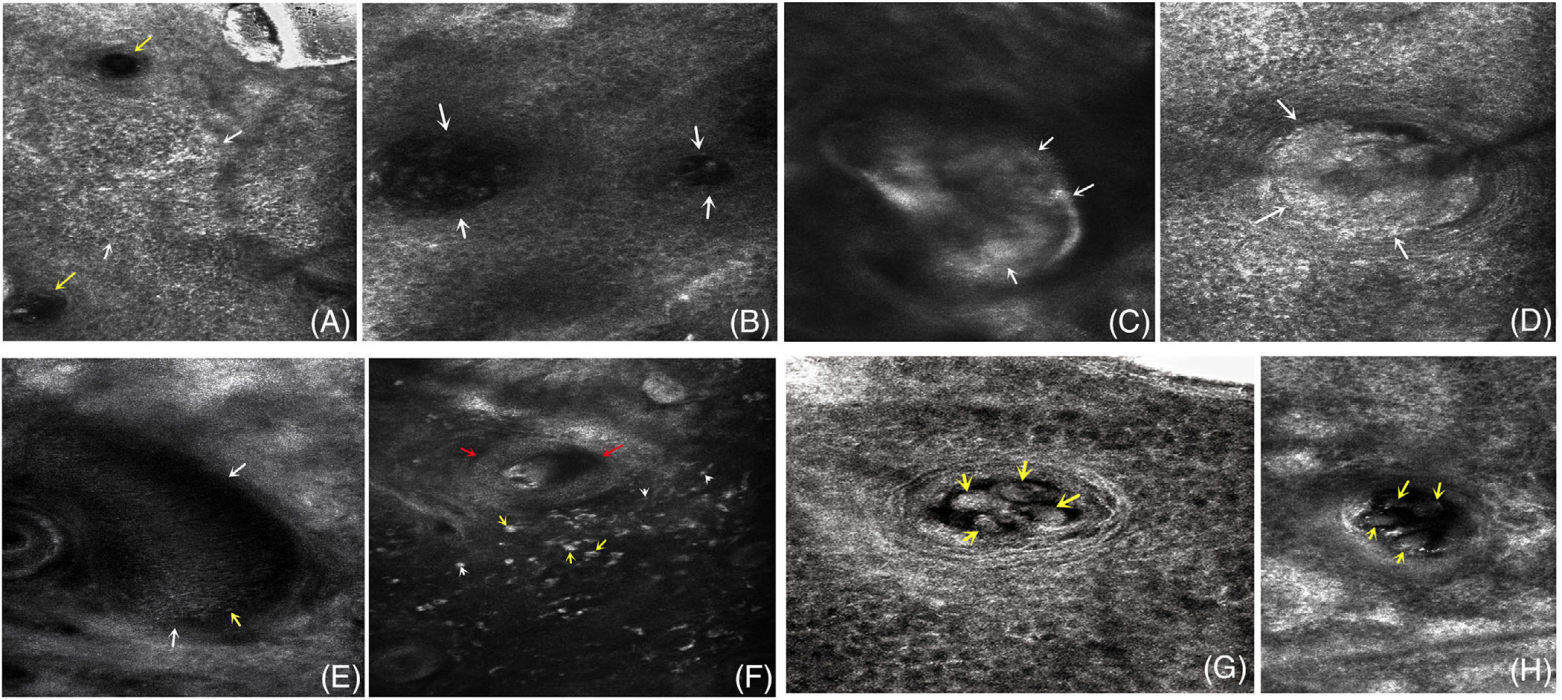
(A) CT scan of the skin showed abrupt changes in the epidermis (white arrow) and hair follicles (yellow arrow); (B) spongy edema in the spinous layer, with low refractive index blisters of different sizes (white arrow); (C) the infundibulum of hair follicle is dilated and filled with high refraction horny like material (white arrow); (D) abscess formation around the hair follicle. High refraction neutrophils can be seen gathering (white arrow) in a vortex shape; (E) the blood vessels around the hair follicle are obviously proliferated and dilated (white arrow), and a large number of low and medium refraction blood cells can be seen (yellow arrow); (F) hair follicle horn plug (red arrow), more inflammatory cells (white arrow) can be seen in the shallow middle layer of dermis and around hair follicles, and some cells are large (yellow arrow); (G) Demodex structure (yellow arrow) can be seen in hair follicle.

Through the previous feature analysis, we have basically mastered the main skin CT features of patients with rose acne, which will provide some objective imaging basis for the correct diagnosis of rose acne in the future. In addition, through the previous analysis, it also aroused some thoughts.

First, the epidermis of patients with rosacea is relatively thin. At this time, under skin CT, it shows that the epidermis shrinks and flattens, the epidermal process disappears, and the true epidermis is very mild, that is, no obvious dermal papillary ring can be seen (Figure 2A). In addition, a large number of medium and high refraction cell particles can sometimes be seen in the shallow and middle dermis of some patients with rosacea, and some of them are large (Figure 3A). At this time, when analyzing the CT characteristics of their skin, it is possible to mistake the previous manifestations for basal cell liquefaction and degeneration (Figure 3B) (the manifestations of the two are very similar under the microscope), so as to misdiagnose rosacea as some other diseases with interface changes, such as lupus erythematosus, sometimes combined with the characteristics of hair follicle angle plug (Figure 3C), collagen fiber hyperplasia in the shallow and middle dermis (Figure 3D) (which may occur in the dermatophyte stage of rosacea). It may also be misdiagnosed as sclerosing atrophic moss and other diseases. Therefore, when we diagnose rosacea, we must combine the clinical manifestations with the skin CT features and make a comprehensive analysis to avoid misdiagnosis. At the same time, we should not give the diagnosis only by relying on a few skin CT features but also find other features that are helpful for differential diagnosis.

**FIGURE 3:**
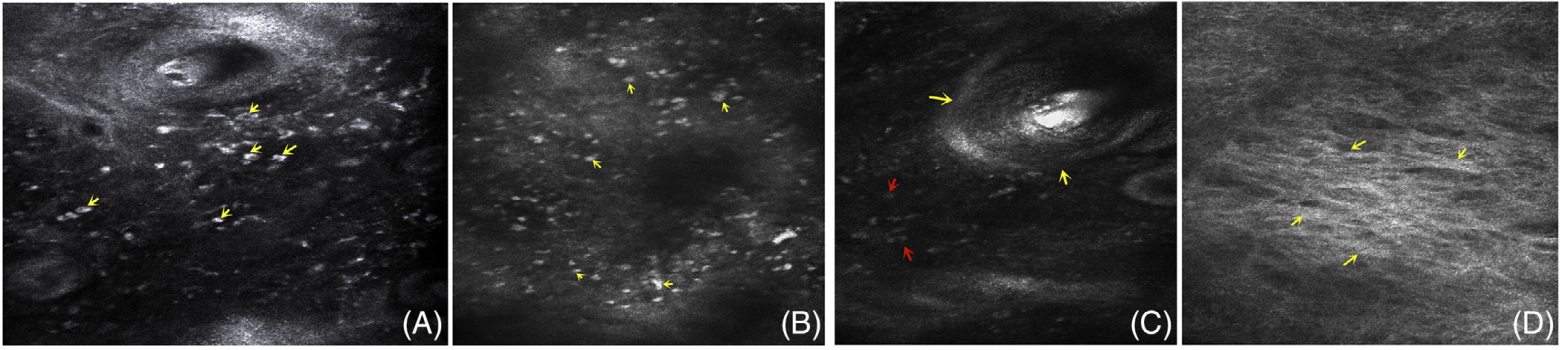
(A) There are many high refraction cell particles (yellow arrow) around the hair follicle of rosacea and the shallow and middle layer of dermis; (B) CT image of skin with typical basal cell liquefaction and degeneration: a large number of high refraction phagocytic pigment cells and inflammatory cell infiltration (yellow arrow); (C) hair follicle horn plug of rose acne (yellow arrow) and scattered medium and high refraction cells around it (red arrow); (D) dermal collagen fiber hyperplasia (yellow arrow) can be seen on CT images of skin in rhinophyma stage of rosacea.

Second, rosacea is a chronic inflammatory skin disease mainly involving the face. Compared with other parts, the skin CT characteristics of facial skin have certain particularity. For example, the epidermal mutation level mentioned in the previous content is almost common and not specific because the facial skin is relatively thin and tender. In addition, the face belongs to the exposed part. If the daily sunscreen is not done well, it is easy to be infringed by ultraviolet rays and form pigmentation. Therefore, through skin CT, it may be seen that there is a large infiltration of pigment phagocytes in the shallow and middle layers of dermis (Figure 4A), and sometimes dendritic cells may appear at the junction of true epidermis (Figure 4B). Therefore, when dendritic cells or large high refraction pigment phagocytes appear in the skin CT manifestations of patients with rosacea, it may be caused by ultraviolet irradiation.

**FIGURE 4:**
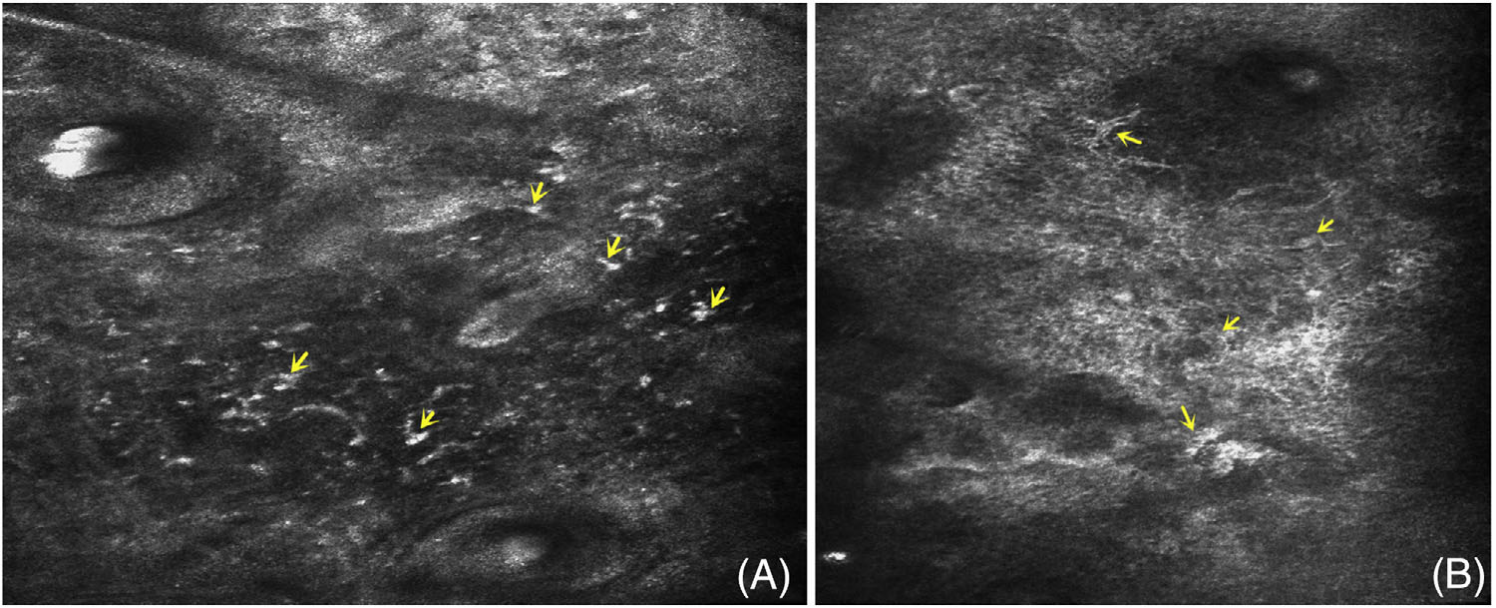
(A) The CT image of rosacea skin shows that there are many large high refraction cell particles infiltrating around the hair follicle and the shallow and middle layer of dermis (yellow arrow); (B) dendritic cells (yellow arrow) can be seen in some rosacea.

Third, the facial inflammation of patients with rosacea is generally serious, and some are accompanied by itching, burning and other symptoms, so there will be certain scratching stimulation, and these stimuli may show more and slightly larger inflammatory cell infiltration under skin CT. This feature has been verified in patients with cervical neurodermatitis. Due to the continuous scratching stimulation of patients with neurodermatitis, we found that the CT features of the skin were scattered infiltration of high refractive cells in the dermal papilla, and some cells were large (Figure 5A). In addition to the previous effects of ultraviolet radiation, it is not uncommon for patients with rosacea to have a large number of high refractive cell particles infiltrating in the superficial and middle dermis. Moreover, we speculate that some large high refraction cell particles (Figure 5B) may correspond to plasma cells in rose acne histopathology. This is of high value for the diagnosis of rose acne.

**FIGURE 5:**
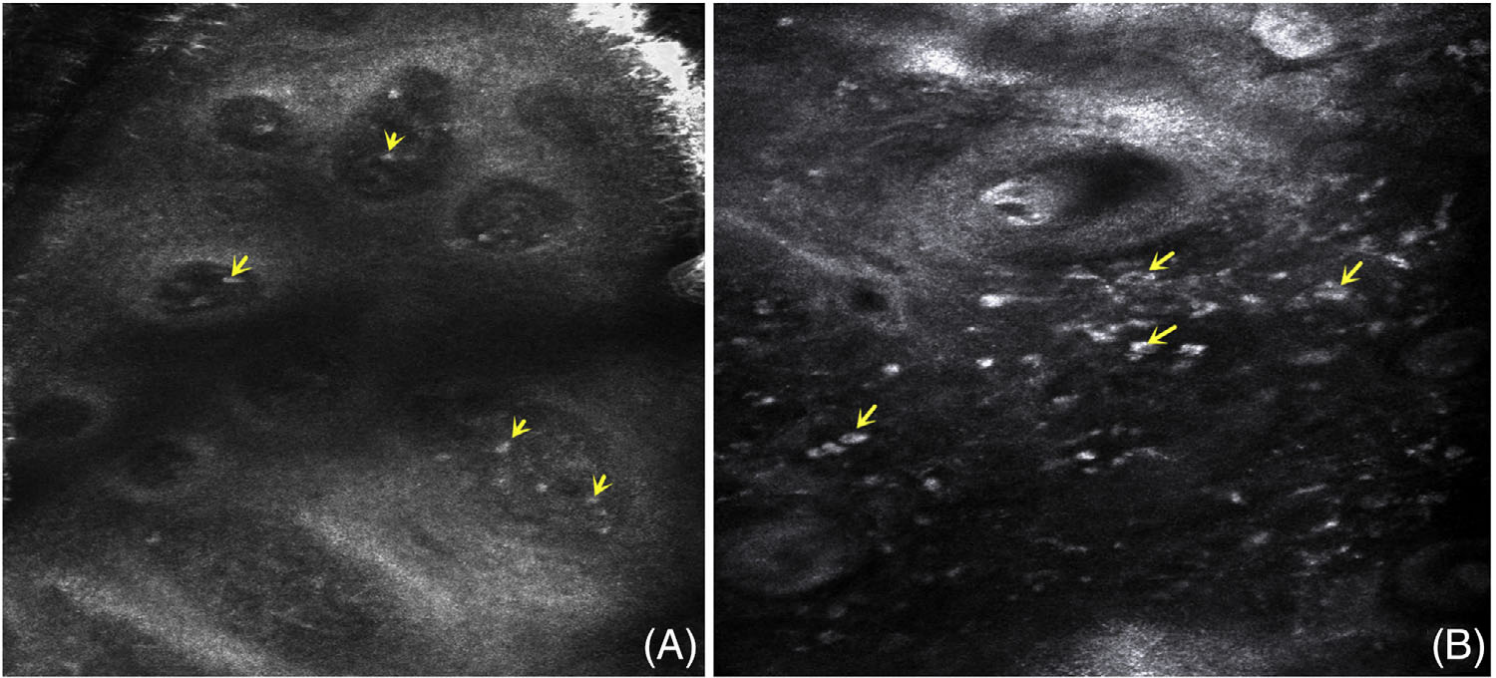
(A) The CT image of neurodermatitis skin shows that there are many high refraction cells (yellow arrow) in the dermal papilla, which may be related to repeated scratching stimulation; (B) sometimes large high refraction cells can be seen in the superficial and middle dermis of rosacea, showing “target type” (yellow arrow), which may correspond to the plasma cells of histopathology.

Through the earlier contents, we hope that we can basically master the skin CT features of rose acne and comprehensively analyze some confusing features to avoid misdiagnosis. Of course, some contents need to be further confirmed. I hope more clinicians will participate in our exploration. I hope this sharing is beneficial to clinicians. Thank you!

## References

[1] Meng Rusong , Cui Yong . Multimodal Dermatology Medical Image Diagnosis Atlas. 1st ed. People's Health Publishing House; 2021:407‐409.

[2] Liu Huaxu . Reflection Confocal Microscope Dermatology Atlas. 1st ed. People's Health Publishing House; 2013:4‐5.

[3] Holmes AD . Potential role of microorganisms in the pathogenesis of rosacea. J Am Acad Dermatol. 2013;69(6):1025‐1032. 10.1016/j.jaad.2013.08.006 24011460

